# Bladder cancer cell‐intrinsic PD‐L1 signals promote mTOR and autophagy activation that can be inhibited to improve cytotoxic chemotherapy

**DOI:** 10.1002/cam4.3739

**Published:** 2021-02-24

**Authors:** Deyi Zhang, Ryan M. Reyes, Erica Osta, Suresh Kari, Harshita B. Gupta, Alvaro S. Padron, Anand V. R. Kornepati, Aravind Kancharla, Xiujie Sun, Yilun Deng, Bogang Wu, Ratna Vadlamudi, Rong Li, Robert S. Svatek, Tyler J. Curiel

**Affiliations:** ^1^ Department of Medicine University of Texas Health San Antonio TX USA; ^2^ Graduate School of Biomedical Sciences University of Texas Health San Antonio TX USA; ^3^ Department of Microbiology, Immunology and Molecular Genetics University Texas Health San Antonio TX USA; ^4^ Mays Cancer Center, University of Texas Health San Antonio TX USA; ^5^ Department of Molecular Medicine University of Texas Health San Antonio TX USA; ^6^ Department of Obstetrics and Gynecology University of Texas Health Science Center San Antonio TX USA; ^7^ Department of Urology University of Texas Health Science Center San Antonio TX USA; ^8^ Present address: National Institutes of Health Bethesda MD USA; ^9^ Present address: Department of Biochemistry & Molecular Medicine School of Medicine & Health Sciences The George Washington University Washington DC USA

**Keywords:** autophagy, bladder cancer, chemotherapy, mTOR, PD‐L1

## Abstract

Tumor cell‐intrinsic programmed death‐ligand 1 (PD‐L1) signals mediate immunopathologic effects in breast, colon, and ovarian cancers and in melanomas, but bladder cancer (BC) effects are unreported. We show here that BC cell‐intrinsic PD‐L1 signals in mouse MB49 and human RT4, UM‐UC3, and UM‐UC‐14 BC cells regulate important pathologic pathways and processes, including effects not reported in other cancers. α‐PD‐L1 antibodies reduced BC cell proliferation in vitro, demonstrating direct signaling effects. BC cell‐intrinsic PD‐L1 promoted mammalian target of rapamycin complex 1 (mTORC1) signals in vitro and augmented in vivo immune‐independent cell growth and metastatic cancer spread, similar to effects we reported in melanoma and ovarian cancer. BC cell‐intrinsic PD‐L1 signals also promoted basal and stress‐induced autophagy, whereas these signals inhibited autophagy in melanoma and ovarian cancer cells. BC cell‐intrinsic PD‐L1 also mediated chemotherapy resistance to the commonly used BC chemotherapy agents cis‐platinum and gemcitabine and to the mTORC1 inhibitor, rapamycin. Thus, BC cell‐intrinsic PD‐L1 signals regulate important virulence and treatment resistance pathways that suggest novel, actionable treatment targets meriting additional studies. As a proof‐of‐concept, we showed that the autophagy inhibitor chloroquine improved cis‐platinum treatment efficacy in vivo, with greater efficacy in PD‐L1 null versus PD‐L1‐replete BC.

## Graphical abstract

We provide evidence that tumor‐intrinsic PD‐L1 mediates pathologic signals in human and mouse bladder cancers, independent of molecular subtype. Signals include control of proliferation, mTORC1, autophagy, and chemotherapy resistance. We show that countering the pathologic cell‐intrinsic PD‐L1 signals improves chemotherapy response in vivo of mice bearing bladder cancer tumors.

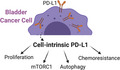

## 1 INTRODUCTION

Programmed death‐ligand 1 (PD‐L1) is an immune cosignaling molecule that negatively regulates T‐cell functions through its known receptors programmed death 1 (PD‐1) and CD80,^1^ and is immunopathogenic in many cancers, including bladder cancer (BC).^2^, ^3^, ^4^ The immune checkpoint blockade immunotherapies anti (α)‐PD‐L1 and α‐PD‐1 have demonstrated clinical efficacy against muscle‐invasive and metastatic BC^5^, ^6^, ^7^, ^8^ and the α‐PD‐L1 antibody pembrolizumab was recently approved for treating nonmetastatic, high‐risk nonmuscle‐invasive BC.^9^ The existing dogma is that α‐PD‐1 and α‐PD‐L1 antibodies protect PD‐1^+^ antitumor T cells from inhibition by tumor surface PD‐L1,^10^ but many mechanistic details of PD‐L1/PD‐1 signaling remain incompletely understood. Further, complete clinical responses to FDA‐approved BC immunotherapies occur in <30% of BC patients for poorly understood reasons.^11^

Though cell‐extrinsic interactions between tumor PD‐L1 and T‐cell PD‐1 are well known to suppress antitumor immunity,^10^ tumor cell‐intrinsic PD‐L1 signals regulate other key biologic functions across multiple tumor types.^12^ We previously reported that tumor cell‐intrinsic PD‐L1 drives tumor mammalian target of rapamycin complex 1 (mTORC1) signals and inhibits autophagy that affected small molecule mTORC1 or autophagy inhibitors^13^ and promoted tumor‐initiating cell generation and function^14^ in melanoma and ovarian cancer. Here we show tumor cell‐intrinsic PD‐L1 mediates important signals and pathologic pathways in BC cells including increasing mTORC1 activation and autophagy, promoting immune‐independent cell growth and metastatic tumor spread, and resistance to cytotoxic chemotherapies and targeted small molecules, in addition to other pathologic effects. Some BC cell‐intrinsic PD‐L1 signals were similar to those in melanoma and ovarian cancer cells, whereas others differed significantly. We used effects of BC cell‐intrinsic PD‐L1 signals to define a novel treatment strategy to improve cis‐platinum treatment efficacy, which showed a greater effect in PD‐L1‐null versus PD‐L1‐replete BC tumors. Other findings described here could be used to improve BC treatments including gemcitabine or cis‐platinum, or immune checkpoint blockade immunotherapies, and could help explain the incomplete predictive power of BC PD‐L1 for α‐PD‐L1 or α‐PD‐1 immunotherapy efficacy.

## 2 MATERIALS AND METHODS

### 2.1 Mice

Wild‐type (WT) C57BL/6 J (BL6) and NOD.Cg‐Prkdc^scid^Il2rg^tm1Wj1^/SzJ [nonobese diabetic/severe combined immunodeficiency (NOD/SCID)/IL2Rγ KO, NSG] mice were purchased from Jackson Laboratory, maintained under specific pathogen‐free conditions and given food and water ad libitum. Age‐ and sex‐matched mice at least 8 weeks old were used. All studies were approved by our Institutional Animal Care and Use Committee.

### 2.2 Tumor cells

**FIGURE 1:**
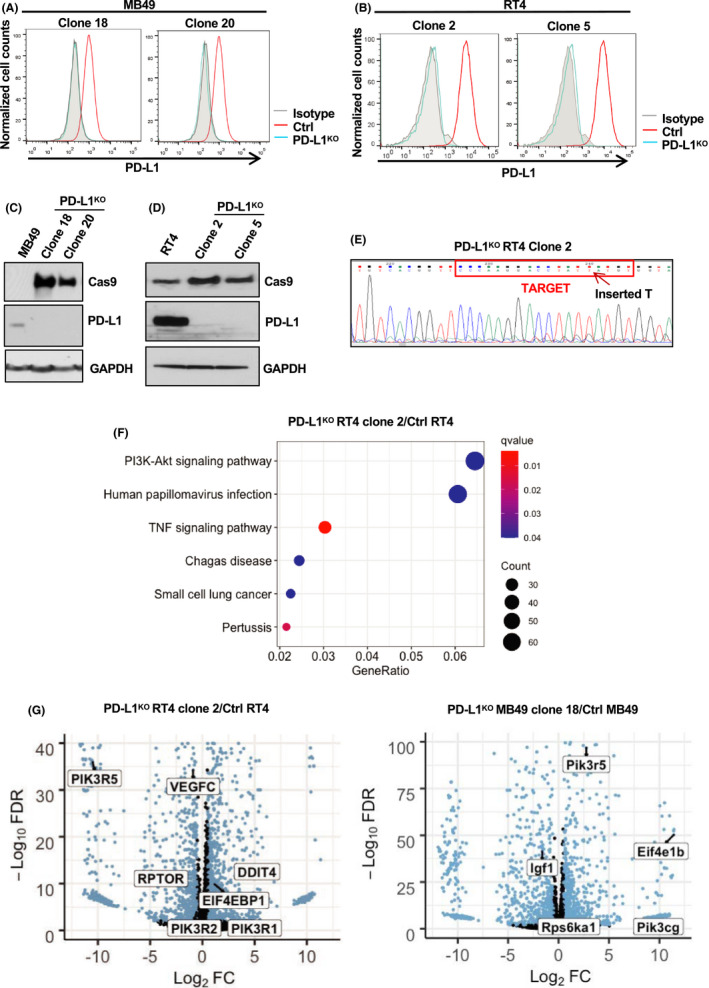
BC cell PD‐L1 expression and PD‐L1^KO^ clones. PD‐L1 was knocked out of BC cell lines by CRISPR/Cas9 and validated using flow cytometry staining (A, B), western blot (C, D), and DNA sequencing (E) of the PD‐L1 Cas9 insertion region. RNA‐seq from PD‐L1^KO^ and control cells grown in vitro. (F) Top KEGG‐enriched pathways in PD‐L1^KO^ compared to control cells. *N* = 3 biologically independent, sequenced samples per group. Dot size indicates the counts of differentially expressed genes enriched per pathway and color indicates q‐value. (G) Volcano plots of differentially expressed genes depicting key genes involved in mTOR signaling pathway. KEGG, Kyoto Encyclopedia of Genes and Genomes. FDR, false discovery rate. FC, fold change

Mouse MB49 BC and human HEK293 T cells (provided by R. Li) were purchased from the American Type Culture Collection. RT4, a human epithelial BC cell line, was provided by R. Svatek. We generated PD‐L1 knockout (PD‐L1^KO^) MB49 using CD274 sgRNA clustered regularly interspaced short palindromic repeats (CRISPR)/Cas9 All‐in‐One Lentivector (mouse) (ABM Cat# 155161140595) that contains three plasmids targeting PD‐L1: (1) 69 TCCAAAGGACTTGTACG; (2) 186 GCAAGTGATTCAGTTTG; and (3) 337 TGCTGCATAATCAGCTA. PD‐L1^KO^ RT4 cells were generated using the human version of the same Lentivector (ABM Cat# 155161110595) that contained three plasmids targeting PD‐L1: (1) 69 TCCCAAGGACCTATATG; (2) 233 ATAGTAGCTACAGACAG; and (3) 337 CGCTGCATGATCAGCTA. HEK293 T cells were cotransfected with pMD2.G and psPAX2 (Addgene) PD‐L1 targeting plasmid, and pLenti‐U6‐sqRNA‐SFFV‐Cas9‐2A‐Puro to generate the lentivirus. Two days after transfection, medium from packaging cells was used to transfect MB49 or RT4 cells with 10 mg/mL Polybrene (Millipore, TR‐1003‐G) at a final concentration of 0.8 μg/mL. Selection was with 2 μg/mL puromycin. Single cells were seeded in a 96‐well plate (100 μL/well), selected using microscopy, and verified using DNA sequencing, western blot, and flow cytometry (Figure 1). The polyclonal PD‐L1 knockdown UM‐UC‐3 and UM‐UC‐14 stable cell lines (PD‐L1^lo^) were generated using lentiviral transduction particles containing prevalidated PD‐L1 shRNA (Sigma) against human *CD274* or a scrambled control shRNA and selected using puromycin as we previously described.^13^ All cell lines were negative for *Mycoplasma* in periodic testing using a MycoAlert Mycoplasma Detection Kit (Lonza, Cat# LT07‐318), according to manufacturer's directions.

Cells were used in passages <5 and maintained in 5% fetal bovine serum (FBS)‐containing DMEM (Dulbecco's modified Eagle medium, MB49) or RPMI‐1640 (Roswell Park Memorial Institute, RT4), supplemented with 1% penicillin/streptomycin, 1% L‐glutamate, and 1% 4‐(2‐hydroxyethyl)‐1‐piperazineethanesulfonic acid (HEPES).

### 2.3 Cell proliferation and viability assays

Cells (6 × 10^3^) were plated in 96‐well plastic culture plates in 100 μL of their respective medium (DMEM for MB49 and RPMI‐1640 for RT4) and treated 12 h later with chloroquine (Selleckchem, Cat# S4157) or rapamycin (Sigma, Cat# R8781‐200UL) at indicated doses and time intervals. Anti‐mouse (10F.9G2, BioXCell, Cat# BE0101) or human (29E.2A3, BioXCell, Cat# BE0285) α‐PD‐L1 antibodies or respective isotype (mouse‐ rat IgG2b, LTF‐2, BioXCell, Cat# BE0090 and human‐ mouse IgG2b, MPC‐11, BioXCell, Cat# BE0086) controls were added at culture initiation at 50 μg/mL. Data shown are from optimized drug concentrations in preliminary work not shown.

For serum starvation experiments, cells were washed with PBS (phosphate‐buffered saline) two times and cultured in EBSS (Earle's Balanced Salt Solution, Gibco, Cat# 24010043) for 4 h. After 4 h, EBSS was replaced with DMEM and cells were treated with chloroquine (20 μM) or 3‐methyladenine (3‐MA) (10 mM, Selleckchem, Cat# S2767) for 6 h.

Cell viability was determined by MTT (3‐(4,5‐dimethylthiazol‐2‐yl)‐2,5‐diphenyltetrazolium bromide) assay. Six thousand cells (MB49) or 4000 cells (RT4) were seeded into a 96‐well plate and cultured for 72 h prior to treatment with MTT (20 μL, 5 mg/ml) in 100 μL medium. A 48‐hour drug incubation period preceded MTT for cis‐platinum (Selleckchem, Cat# S1166) or gemcitabine (Selleckchem, Cat# S1149) incubations. Cells were incubated at 37°C for 4 h with MTT. Absorbance was measured at 540 nm using a BioTek Synergy 2 Multi‐Mode Plate Reader. Proliferation was assessed in triplicate in three separate experiments. MTT data were validated using actual cell counts on a Vi‐cell XR Cell Viability Analyzer (Beckman Coulter) in all experiments.

### 2.4 In vivo tumor challenges, treatments, and assessments

2 × 10^6^ RT4 cells/flank or 2 × 10^5^ MB49 cells/flank were given subcutaneously (both sexes). 8 × 10^4^ MB49 cells were given intravesically (in bladder, females only, under ketamine/xylazine anesthesia^15^). Subcutaneous tumor growth was measured with Vernier calipers and volume was calculated as (length × width^2^)/2. Orthotopic tumor growth was determined by bladder weight. Survival was determined as ≥1500 mm^3^ for subcutaneous tumors and distress or >20% mouse weight loss from baseline.^16^ Intraperitoneal injection of chloroquine (60 mg/kg) and cis‐platinum (2 or 3 mg/kg) was given every 2–3 days starting on day 7 post tumor challenge, as indicated.

### 2.5 Flow Cytometry

Cells were stained as previously described,^17^ using LSR II hardware and analyzed by FACSDiva (BD Bioscience) or FlowJo software (BD Bioscience). Anti‐mouse (10F.9G2) and human (29E.2A3) PD‐L1 (both PE/Cy7, Cat# 124314 [mouse] 329718 [human]), Ki67 (APC, Cat# 350514), and isotype control antibodies and were purchased from BioLegend. Cells were counted with a Vi‐cell. Apoptosis assessment used Annexin V and propidium iodide (PI) (Thermo Scientific) per manufacturer specifications.

### 2.6 Immunoblotting

Immunoblotting was performed according to a previously published protocol.^13^ Briefly, cell lysates were prepared in RIPA (Radioimmunoprecipitation assay) buffer (20 mM Tris–HCl pH 8.0, 150 mM NaCl, 1 mM disodium EDTA, 1 mM EGTA, 2.5 mM sodium pyrophosphate, 1 mM β‐glycerophosphate, 1% Triton X‐100) plus 1 mM phenylmethylsulphonyl fluoride and Halt protease/phosphatase inhibitor cocktail (Thermo Scientific). Protein concentration was measured by Bradford assay (Thermo Scientific). 20–30 μg of protein was run on precast 4–15% sodium dodecyl sulfate polyacrylamide gels (Bio‐Rad), transferred to polyvinylidene fluoride membranes (GE Water and Process Technologies), blocked in Tris‐buffered saline (pH 7.4) plus 0.1% Tween‐20 and 5% skim milk, and incubated overnight at 4°C with 1:1000 diluted antibodies against indicated proteins (Cell Signaling) plus anti‐mouse GAPDH (Santa Cruz Biotechnology) or Vinculin (Cell Signaling) as loading controls. Membranes were incubated with species‐appropriate horse radish peroxide‐conjugated secondary antibodies for 1–2 h. Proteins were detected by enhanced chemiluminescence using Western Lightening Plus reagent (Perkin Elmer). Band quantification and normalization to total protein was by ImageJ software.^18^ Data are shown as means of three individual blots with comparisons only made between like blots from the same gels.

### 2.7 Microscopy

Cells were plated at low confluence in six‐well plates (50,000 cells/well). The following day, cells were exposed to serum starvation (EBSS), normal medium (10% FBS), or chloroquine (20 µM) for 6 h. Medium was removed, cells were washed with PBS and treated with 4% paraformaldehyde/PBS for 20 min at room temperature, washed, then permeabilized with 0.1% Triton X‐100 for 10 min. Cells were then blocked with 5% normal goat serum (Cell Signaling Technology) containing 0.3% Triton X‐100 in PBS for 1 h. Diluted (1:200) anti‐mouse microtubule‐associated protein 1A/1B‐light chain 3 (LC3) A/B (Cell Signaling Technology) was applied in blocking buffer overnight at 4°C. Alexa Fluor‐555 secondary antibody diluted in 1% normal goat serum in PBS was added for 1 h at ambient temperature. Cells were fixed using Vectashield hard set mounting medium containing DAPI (4’,6 ‐diamidino‐2‐phenylindole, Vector Laboratories). Images were acquired using confocal microscopy (Olympus FV‐1000) and overlaid using ImageJ.^18^

### 2.8 Rna‐seq and bioinformatics analyses

Total RNA from control or PD‐L1^KO^ MB49 and RT4 cells was purified using RNeasy (Qiagen), and RNA quality was ensured on an Agilent Bioanalyzer. Fifty‐base‐pair single read sequencing was performed using Illumina HiSeq 2000 at the Genome Sequencing Facility at UTHSA. Differential gene expression analysis was performed using R package DESeq2. Top enriched Kyoto Encyclopedia of Genes and Genomes (KEGG) pathways were determined with R packages clusterProfiler and enrichplot (Figure 1F) ranked by q‐value (Table 1). Individual differentially expressed genes in PD‐L1^KO^/control cells were plotted using R package EnhancedVolcano (Figure G). Next generation sequencing (NGS) data will be deposited in the Gene Expression Omnibus (GEO) database.

**TABLE 1:** Top enriched terms from KEGG pathway enrichment of differentially expressed genes shown as PD‐L1^KO^/control ranked by q‐value

PD‐L1^KO^ MB49 clone 18/Ctrl MB49						
ID	Description	GeneRatio	BgRatio	*p* value	p adjust	q value
mmu04512	ECM‐receptor interaction	22/954	88/8913	1.06E−04	2.62E−02	2.27E−02
mmu04010	MAPK signaling pathway	52/954	294/8913	1.66E−04	2.62E−02	2.27E−02

PD‐L1^KO^ RT4 clone 2/Ctrl RT4						
ID	Description	GeneRatio	BgRatio	*p* value	p adjust	q value
hsa04668	TNF signaling pathway	31/1023	112/8077	1.44E−05	4.59E−03	3.95E−03
hsa05133	Pertussis	22/1023	76/8077	1.21E−04	1.92E−02	1.65E−02
hsa04151	PI3 K‐Akt signaling pathway	66/1023	354/8077	6.47E−04	4.66E−02	4.01E−02
hsa05142	Chagas disease	25/1023	102/8077	7.33E−04	4.66E−02	4.01E−02
hsa05165	Human papillomavirus infection	62/1023	331/8077	8.28E−04	4.66E−02	4.01E−02
hsa05222	Small cell lung cancer	23/1023	92/8077	8.80E−04	4.66E−02	4.01E−02

Abbreviations: ECM, extracellular matrix; MAPK, mitogen‐activated protein kinase; TNF, tumor necrosis alpha; PI3 K, phosphoinositol 3‐kinase.

### 2.9 Statistical analysis

Statistical analyses were conducted with Prism software version 8.0 (GraphPad). Data in bar graphs are means ± SEM. For tumor growth, we used two‐way ANOVA plus Bonferroni posttests to compare replicate means. Kaplan–Meier estimates and the log‐rank test were used to analyze mouse survival. For all other single measurement assays, we used an unpaired *t* test. *p* < 0.05 was considered statistically significant.

## 3 RESULTS

### 3.1 PD‐L1^KO^ clone generation from PD‐L1‐expressing BC cell lines

PD‐L1 is expressed on mouse MB49 (Figure 1A), and human RT4 bladder cancer cells (Figure 1B). We used CRISPR/Cas9 to delete PD‐L1 and validated PD‐L1 knockout by flow cytometry (Figure 1A,B), western blot (Figure 1C,D), and DNA sequencing (Figure 1E). In further confirmation of PD‐L1^KO^ sufficiency, we found that incubating control, but not PD‐L1^KO^ cells with recombinant interferon‐γ significantly increased PD‐L1 mean fluorescence intensity (data not shown). We selected PD‐L1^KO^ MB49 clones 13, 18, and 20 and PD‐L1^KO^ RT4 clones 2 and 5 for additional studies.

### 3.2 Tumor cell‐intrinsic PD‐L1 regulates BC cell gene expression in major, canonical pathways

We used RNA‐seq followed by KEGG pathway analysis to demonstrate that BC cell‐intrinsic PD‐L1 altered genes in many canonical signaling pathways (Figure 1F,G, Table 1). For example, PD‐L1 regulated genes involved in multiple signaling and cytokine pathways such as mitogen‐activated protein kinase, phosphoinositol 3‐kinase‐Akt, and tumor necrosis alpha signaling.

### 3.3 Tumor cell‐intrinsic PD‐L1 promotes human RT4 BC cell proliferation but not mouse MB49 BC cell proliferation in vitro

**FIGURE 2:**
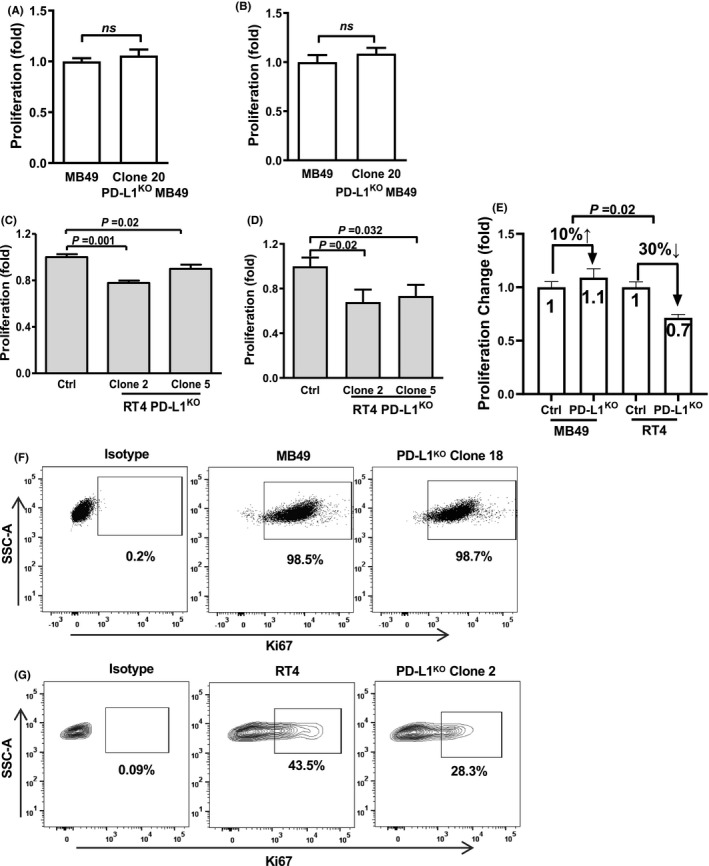
Tumor cell‐intrinsic PD‐L1 alters in vitro BC cell proliferation. MTT viability assay of MB49 (A) and RT4 (C) control and PD‐L1^KO^ cell lines at 72 h. MB49 (B) and RT4 (D) cell counts after control and PD‐L1^KO^ cells were uniformly seeded in 12‐well plates for 72 h. (E) Comparison of BC cell‐intrinsic PD‐L1 effects between cell lines. Flow cytometry staining for Ki67 of MB49 (F) and RT4 (G) cells after 72 h. P, unpaired *t*‐test. SSC‐A, side scatter area

We reported that tumor cell‐intrinsic PD‐L1 promoted in vitro proliferation of mouse melanoma and ovarian cancer cells and human ovarian cancer cells.^13^ PD‐L1^KO^ MB49 cells proliferated similar to control MB49 by MTT assay (Figure 2A), confirmed with actual cell counts (Figure 2B). However, RT4 cell‐intrinsic PD‐L1 promoted cell proliferation by MTT and cell counts (Figure 2C,D), which differed in direction and magnitude compared to MB49 cells (Figure 2E). Baseline Ki67 expression was high in MB49 cells and unaffected in PD‐L1^KO^ cells (Figure 2F), consistent with MTT data. PD‐L1^KO^ RT4 cells expressed lower Ki67 versus control RT4 cells (Figure 2G), consistent with MTT data and cell counts. These data support differential effects of tumor cell‐intrinsic PD‐L1 on proliferation between mouse (MB49) and human (RT4) BC.

### 3.4 α‐PD‐L1 antibody suppresses in vitro BC cell proliferation

**FIGURE 3:**
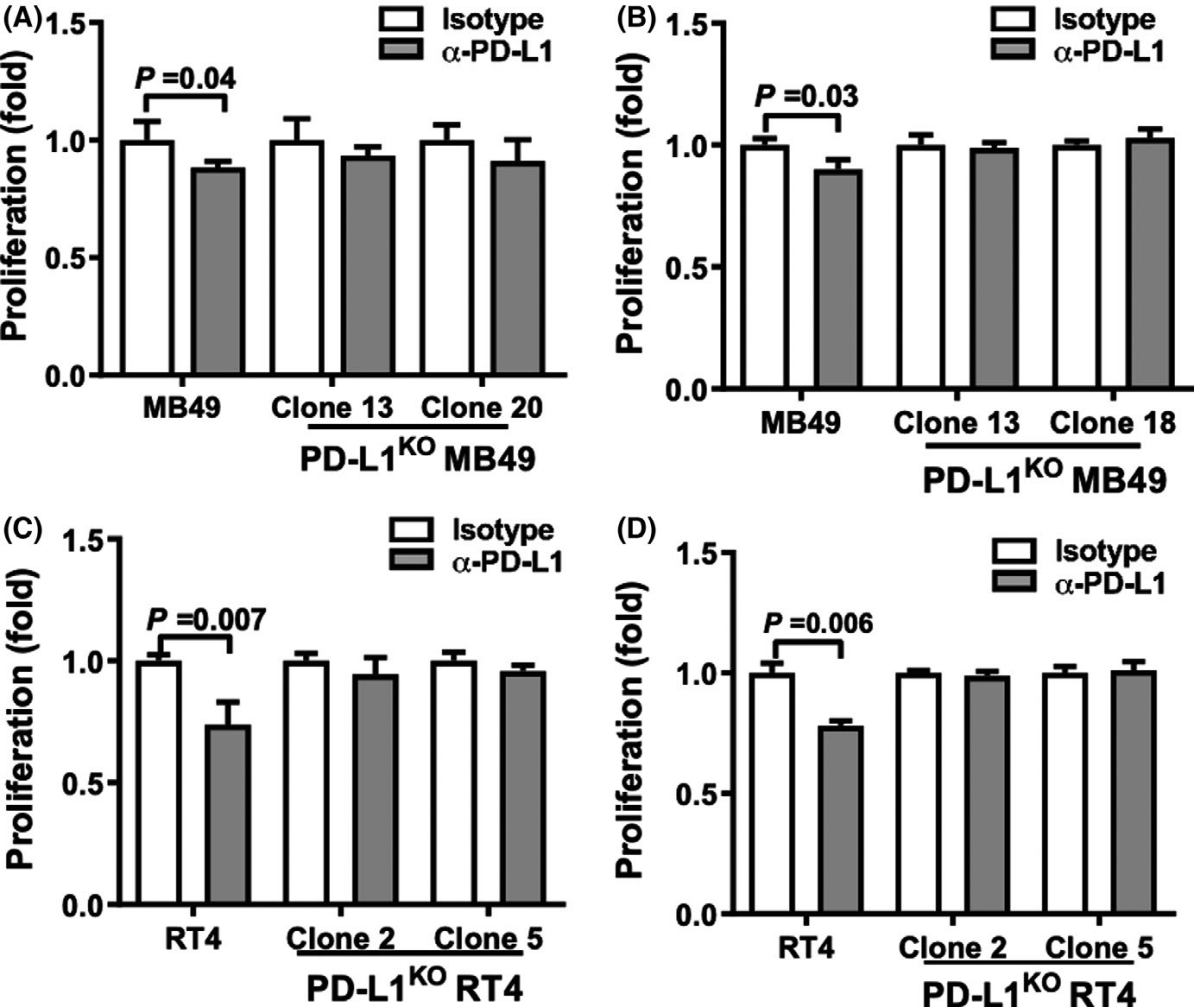
α‐PD‐L1 regulates in vitro BC cell proliferation**.** MTT assay of MB49 (A) and RT4 (C) control and PD‐L1^KO^ cell lines after in vitro culture with α‐PD‐L1 antibody or isotype control (50 μg/mL) for 72 h. MB49 (B) and RT4 (D) cell counts after control and PD‐L1^KO^ cells were uniformly seeded in 12‐well plates and treated with α‐PD‐L1 or isotype control (50 μg/ml) for 72 h. P, unpaired *t*‐test

Although genetic knockout of tumor cell‐intrinsic PD‐L1 did not suppress MB49 proliferation in vitro, α‐PD‐L1 antibody significantly slowed MB49 proliferation in vitro by MTT assay (Figure 3A), which was confirmed by actual cell counts (Figure 3B). Similarly, α‐PD‐L1 slowed control but not PD‐L1^KO^ RT4 cell proliferation in vitro (Figure 3C,D), consistent with our studies in melanoma and ovarian cancer.^13^ PD‐L1^KO^ MB49 and RT4 lines were unaffected by α‐PD‐L1 as expected (Figure 3A‐D). We obtained similar data when cells were treated in medium containing heat‐inactivated serum (data not shown), excluding complement‐dependent cytotoxicity, suggesting unexpected negative signals from α‐PD‐L1 antibody that are absent in PD‐L1^KO^ MB49.

### 3.5 Tumor cell‐intrinsic PD‐L1 controls in vivo BC growth and metastatic spread

**FIGURE 4:**
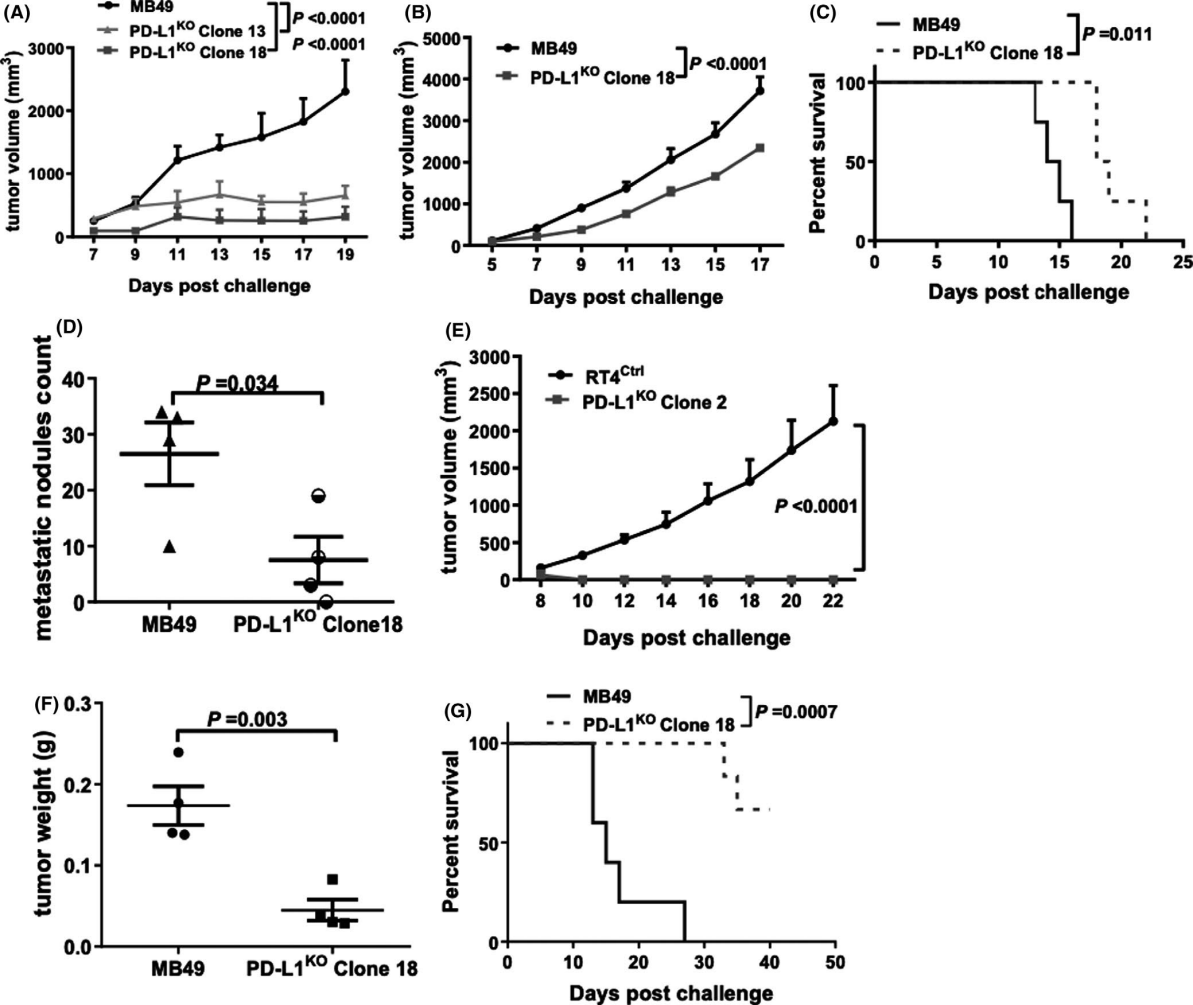
BC cell‐intrinsic PD‐L1 controls tumor growth. (A) Tumor growth in WT mice challenged subcutaneously with 200,000 control or PD‐L1^KO^ MB49 cells. Tumor growth (B), survival (C), and lung metastatic dissemination (D) in NSG mice challenged subcutaneously with 200,000 control or PD‐L1^KO^MB49 cells/flank. (D) Lungs harvested on day 17 post challenge. (E) Tumor growth in NSG mice challenged subcutaneously with 2,000,000 control or PD‐L1^KO^ RT4 cells/flank. Bladder/tumor weight (F) and survival (G) of WT mice challenged orthotopically with 80,000 control or PD‐L1^KO^ MB49 cells. P, (A, B, E) two‐way ANOVA, (D, F) unpaired *t*‐test, (C, G) Kaplan–Meier plot, log‐rank test, (D, F) unpaired *t*‐test

We subcutaneously challenged WT mice with 2 × 10^5^ MB49 cells which induced reliable, robust tumor growth (Figure 4A), consistent with a prior report.^19^ By contrast, subcutaneous challenge of 2 × 10^5^ PD‐L1^KO^ MB49 cells led to poor growth and spontaneous tumor rejection (Figure 4A) and subcutaneous challenge with up to 2 × 10^6^ PD‐L1^KO^ MB49 cells failed to produce reliable tumor growth (Figure S1).

To determine immune contributions to BC PD‐L1‐dependent growth, we challenged severely immune‐deficient NSG mice subcutaneously with control versus PD‐L1^KO^ MB49 cells. In contrast to growth in WT mice, PD‐L1^KO^ MB49 cells grew reliably in NSG mice without regression (Figure 4B). However, PD‐L1^KO^ tumor growth in NSG mice was still significantly slower than control MB49 cells (Figure 4B) and NSG mice challenged with PD‐L1^KO^ MB49 lived significantly longer versus mice hallenged with control MB49 (Figure 4C). NSG mice challenged with PD‐L1^KO^ MB49 cells also had reduced lung metastases compared to control tumor challenged mice (Figure 4D). Strikingly, PD‐L1^KO^ RT4 cells failed to establish subcutaneous tumors in NSG mice although control RT4 cells grew well (clone 2 data, Figure 4E with similar results using clone 5), supported by the in vitro data suggesting that RT4 cells are more dependent on tumor cell‐intrinsic PD‐L1 growth signals versus MB49 cells. Together, these data demonstrate that tumor cell‐intrinsic PD‐L1 exerts immune‐independent pro‐growth effects on mouse and human BC cells in vivo, with in vivo effects far more apparent versus in vitro effects.

To distinguish tumor environment versus tumor cell‐intrinsic PD‐L1 contributions to differential BC growth further, we challenged mice with orthotopic tumors via intravesical inoculation of tumor cells as described^20^ using equal numbers of control versus PD‐L1^KO^ MB49 cells. Bladders from mice challenged with PD‐L1^KO^ MB49 cells weighed significantly less than those of control MB49 challenged mice (Figure 4F) and PD‐L1^KO^ MB49 tumors were less virulent, as evidenced by significantly prolonged survival versus control MB49 challenge (Figure 4G). Control RT4 cells did not establish tumors after orthotopic (bladder) challenge in NSG mice using up to 80,000 cell challenge over 30 days of observation (not shown). Thus, BC cell‐intrinsic PD‐L1 controls in vivo tumor growth distinctly in different tumors and anatomic compartments.

### 3.6 BC cell‐intrinsic PD‐L1 increases mTORC1 signals and AKT^S473^ phosphorylation

**FIGURE 5:**
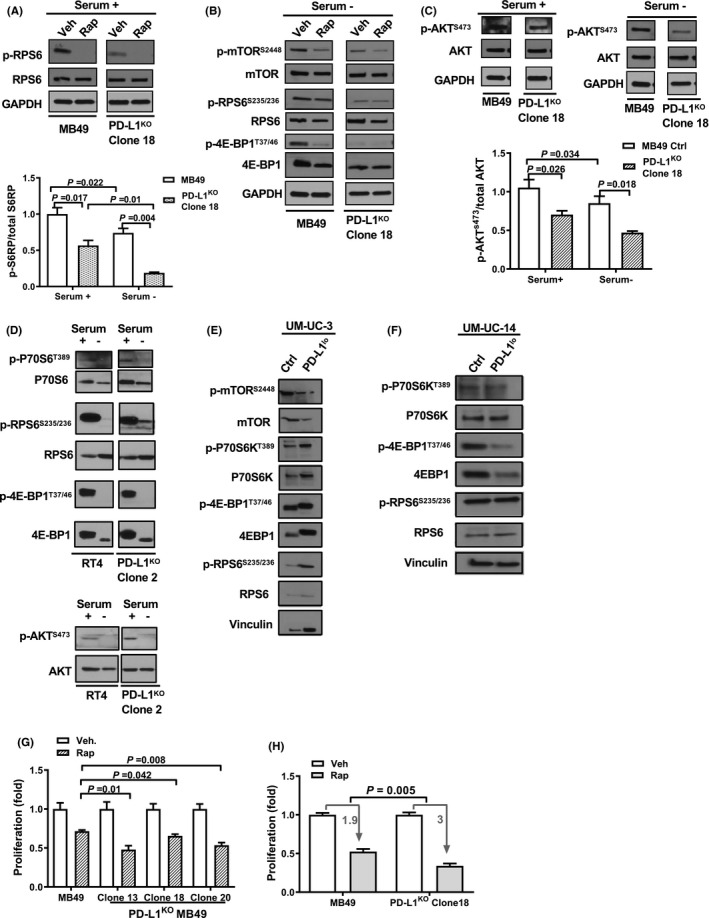
BC cell‐intrinsic PD‐L1 promotes mTOR signals. Western blot of control and PD‐L1^KO^ MB49 cells in serum replete (A, C) and starved conditions (B, C) ±incubation with 2.5 nM rapamycin for 6 h and blots from RT4 and PD‐L1^KO^ RT4 cells in serum replete and starved conditions (D). Western blot of control and PD‐L1^lo^ UM‐UC‐3 (E) and UM‐UC‐14 cells (F) in serum replete conditions. Quantification of selected proteins shown in (A) and (C). All blots were run on the same membranes but separated spatially. Lanes are unedited but have been reorganized for presentation clarity. (G,H) MTT assay of control or PD‐L1^KO^ MB49 cells after treatment with 2.5 nM rapamycin for 72 h. Veh, vehicle. Rap, rapamycin. P, (C‐E,G,H) unpaired *t*‐test

Tumor cell‐intrinsic PD‐L1 promoted basal mTORC1 activity as PD‐L1^KO^ MB49 cells showed decreased activation (phosphorylation) of mTORC1 downstream targets RPS6 and 4E‐BP1 versus control MB49 cells and reduced phosphorylated mTOR (Figure 5A,B), consistent with our prior data from melanoma and ovarian cancer cells.^13^ However, AKT^S473^ phosphorylation was also reduced in PD‐L1^KO^ MB49 cells (Figure 5C), consistent with PD‐L1‐driven mTORC2 activation, in contrast to the increased AKT^S473^ phosphorylation we reported in PD‐L1‐depleted melanoma and ovarian cancer, but similar to PD‐L1 effects in human ES2 ovarian cancer cells.^13^ Serum starvation did not affect PD‐L1‐driven mTORC1 signals but reduced pAKT^S473^ in control and PD‐L1^KO^ MB49 cells (Figure 5B,C) in contrast to effects we reported in melanoma and ovarian cancer^13^ altogether consistent with defective nutrient sensing in these cells. RT4 cells had low basal mTORC1 signals that were reduced further in PD‐L1^KO^ RT4 cells, whereas pAKT^S473^ increased in PD‐L1^KO^ (Figure 5D). Thus, basal and serum starvation PD‐L1‐driven mTORC1 and AKT^S473^ signals differ in distinct BC cell lines, which could also reflect human versus mouse differences.

Muscle‐invasive BC can be categorized into molecular subtypes, helping classify tumors that harbor an array of genetic mutations.^21^ To determine if tumor cell‐intrinsic PD‐L1 mTOR effects were subtype‐dependent, we used PD‐L1 shRNAs to generate PD‐L1^lo^ UM‐UC‐3 (basal‐mesenchymal/claudin‐low)^22^ and UM‐UC‐14 human (luminal)^21^ BC cell lines (Figure S2) for comparison to studies in luminal human RT4 BC.^23^ PD‐L1 depletion markedly reduced mTORC1 activation in both UM‐UC‐3 and UM‐UC14 (Figure 5E,F), suggesting that tumor cell‐intrinsic PD‐L1 mTORC1 activation is common in human BC cell lines irrespective of molecular subtype.

### 3.7 BC cell‐intrinsic PD‐L1 modulates the effects of pharmacologic mTOR inhibition

The mTORC1 inhibitor rapamycin significantly suppressed mTORC1 signals in control and PD‐L1^KO^ MB49 cells (Figure 5A,B). Control and PD‐L1^KO^ MB49 cells were both sensitive to rapamycin‐mediated suppression of proliferation in vitro, but the effect in PD‐L1^KO^ cells was significantly greater (Figure 5G,H), suggesting BC cell‐intrinsic PD‐L1 mediates resistance to rapamycin.

### 3.8 BC cell‐intrinsic PD‐L1 promotes autophagy

**FIGURE 6:**
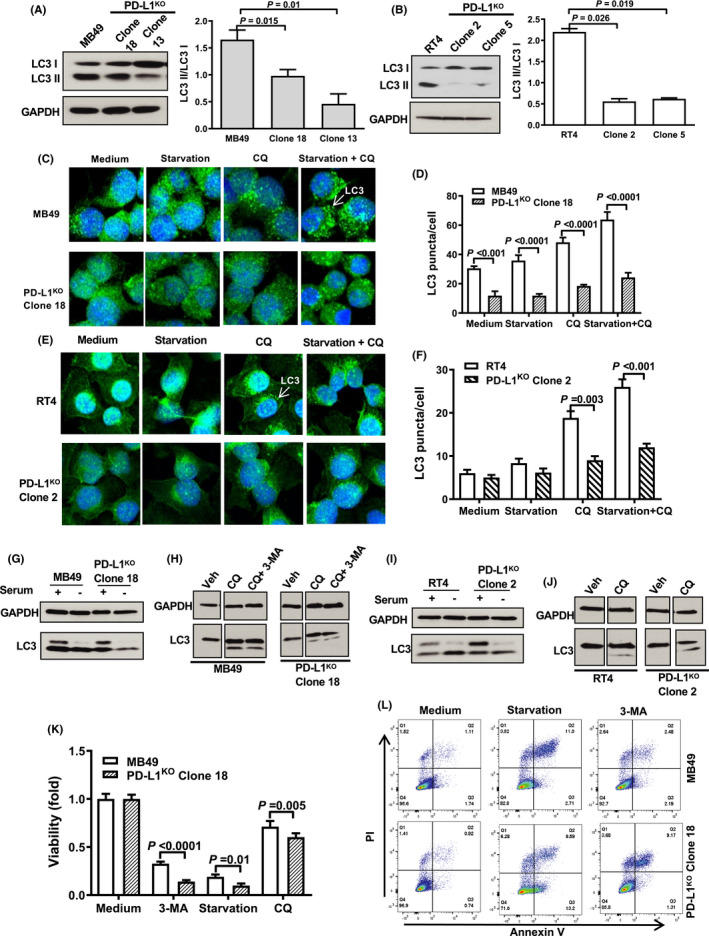
BC cell‐intrinsic PD‐L1 promotes autophagy. (A,B) Western blot showing LC3‐II/LC3‐I ratio in control and PD‐L1^KO^ MB49 (A) and RT4 (B) cells. Immunofluorescence staining and quantification(C‐F) and western blot (G‐J) of LC3 puncta at baseline and in response to starvation conditions (EBSS) or pharmacologic autophagy manipulation (chloroquine and 3‐methyladenine). Starvation conditions and treatments are a 6‐hour incubation in either EBSS medium, 20 μM chloroquine (CQ), or 10 mM 3‐methyladenine (3‐MA). Viability assessment (K, L) in MB49 cells 14 h after shown treatments by flow cytometry for Annexin V and propidium iodide (PI). Veh, vehicle. P, (A,B,D,G,K) unpaired *t*‐test

Because of the clear BC cell‐intrinsic PD‐L1 effects on mTOR signaling, we also examined the role of PD‐L1 in autophagy, a process well known to be linked to mTOR signals.^24^ We found that BC cell‐intrinsic PD‐L1 promotes autophagy, as detected by a reduced LC3‐II/LC3‐I ratio in PD‐L1^KO^ MB49 and RT4 versus respective control cells (Figure 6A,B), in striking contrast to mouse B16 melanoma and ID8agg ovarian cancer cells and human ES2 ovarian cancer cells, where we found that tumor cell‐intrinsic PD‐L1 inhibited autophagy.^13^ In support, we used imaging to detect fewer LC3 puncta in PD‐L1^KO^ versus control cells (Figure 6C‐F). Control cells exhibited a normal autophagy response to starvation with increased LC3 puncta. By contrast, PD‐L1^KO^ MB49 and RT4 cells exhibited an abnormal autophagy response with almost no change in LC3 puncta in starvation and significantly blunted responses versus respective control cells, consistent with their blunted mTORC1 response to serum starvation.

Chloroquine is a pharmacologic autophagy inhibitor and prevents LC3‐II degradation.^25^ 3‐Methyladenine is an autophagy inhibitor that blocks autophagosome formation through phosphoinositol 3‐kinase inhibition and promotes autophagic flux.^26^ Chloroquine alone or in combination with 3‐methyladenine promoted LC3‐II accumulation in control and PD‐L1^KO^ cells (Figure 6C‐J) although cell viability was reduced by both drugs and by starvation (Figure 6K), which was attributable to apoptosis (Figure 6L). Nonetheless, 3‐methyladenine and starvation effects on viability were greater in PD‐L1^KO^ cells (Figure 6K). These data support the concept that both steady‐state autophagy and autophagy flux are promoted by BC cell‐intrinsic PD‐L1.

### 3.9 PD‐L1 decreases BC cell chemotherapy sensitivity

**FIGURE 7:**
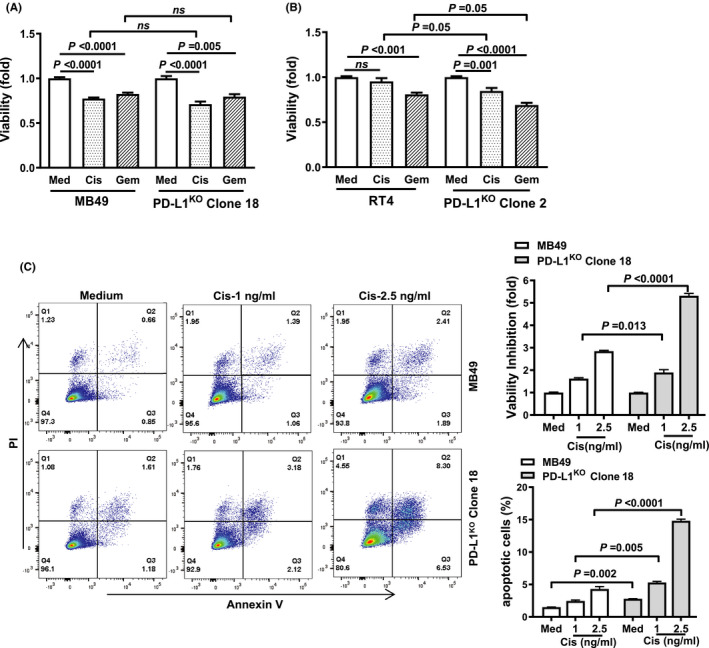
PD‐L1 decreases chemotherapy sensitivity in BC cells. MTT viability assay of control and PD‐L1^KO^ MB49 (A) and RT4 (B) cells. Treatments are a 48‐hour incubation in drug‐free medium, 10 μM cis‐platinum (cis), or 2 ng/mL gemcitabine (gem). Viability assessment (C) in MB49 cells in response to cis‐platinum for 14 h prior to flow cytometry staining for Annexin V and propidium iodide (PI). P, (A‐C) unpaired *t*‐test

Prior work in osteosarcoma, breast cancer, and head and neck cancer models have demonstrated that tumor PD‐L1 expression predicts drug chemotherapy resistance.^27^, ^28^, ^29^ We directly tested the effect of PD‐L1 on chemotherapy sensitivity by assessing cytotoxicity of in vitro treatment with the commonly used BC chemotherapies, cis‐platinum and gemcitabine. Control and PD‐L1^KO^ MB49 cells were similarly sensitive to both agents (Figure 7A), yet RT4 cells were only sensitive to gemcitabine (Figure 7B). PD‐L1^KO^ in RT4 cells rendered them cis‐platinum sensitive, with PD‐L1^KO^ RT4 cells showing increased sensitivity to both agents compared to control RT4 cells. Cis‐platinum also differentially affected PD‐L1^KO^ MB49 cells, as increasing doses of cis‐platinum had a much more significant apoptotic effect on PD‐L1^KO^ MB49 compared to control cells (Figure 7C).

### 3.10 Chemotherapy and autophagy inhibitors combine to treat BC in vivo in a PD‐L1‐dependent manner

**FIGURE 8:**
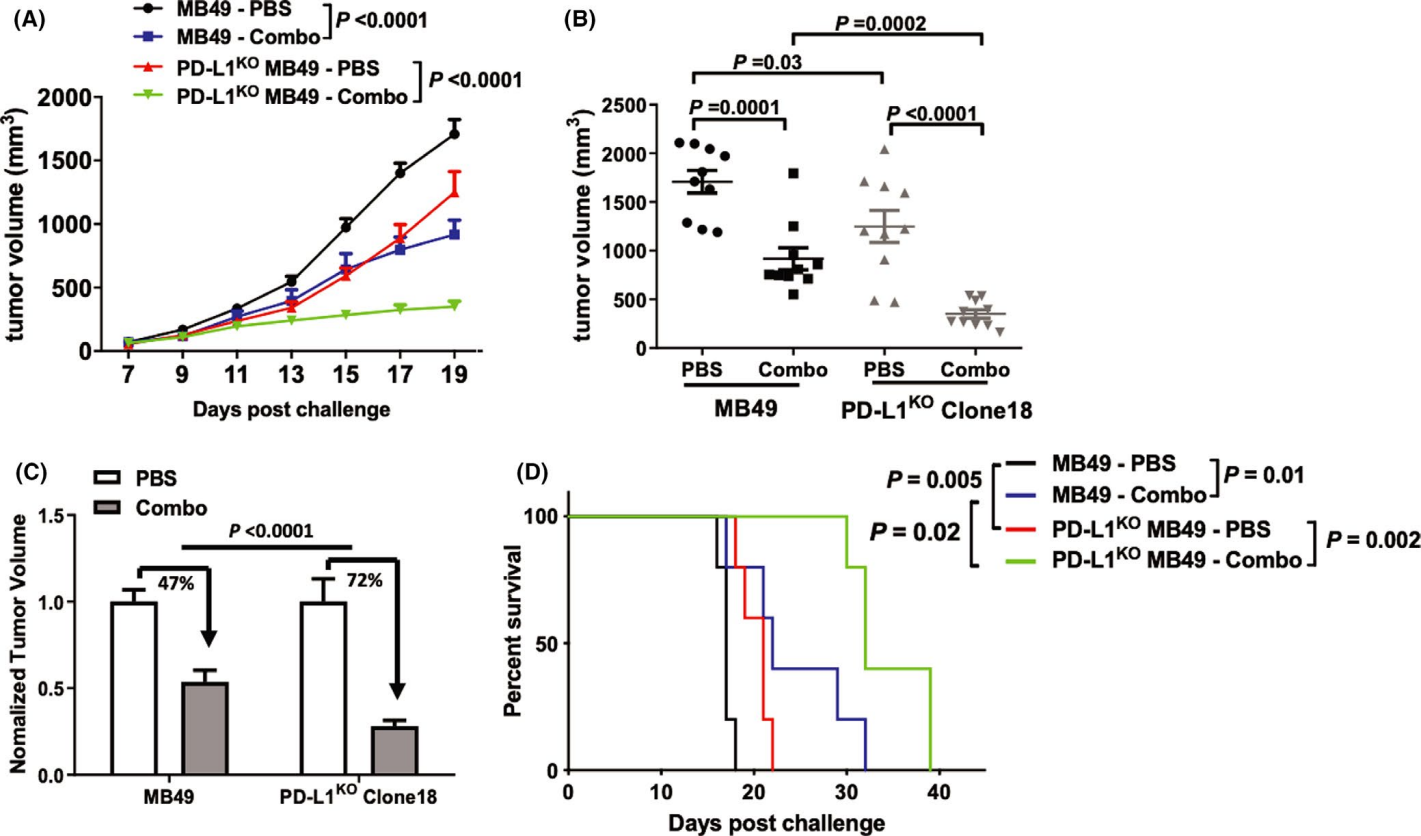
Chloroquine plus cis‐platinum treats PD‐L1^KO^ MB49 better than control MB49 BC. Male NSG mice were challenged subcutaneously with 200,000 MB49 or PD‐L1^KO^ MB49 (clone 18) cells/flank and treated with PBS or combination therapy (cis‐platinum + chloroquine). Cis‐platinum dose was 3 mg/kg on day 7, 9, 11, 13 and 2 mg/kg on day 15, 17, 19. Chloroquine dose was 60 mg/kg on day 7, 10, 12, 14, 16, 18. Absolute (A,B) and relative (C) tumor growth was compared within and between groups. (B,C) Comparison of tumor measurements on day 19 post challenge. (D) Mice were sacrificed at tumor size ≥1500 mm^3^. P, (A) two‐way ANOVA, (B,C) unpaired *t*‐test, (D) Kaplan–Meier plot, log‐rank test

Control versus PD‐L1^KO^ RT4 cells could not be tested in vivo for treatment effects, as PD‐L1^KO^ RT4 cells did not establish tumors in NSG mice. The combination of cis‐platinum plus chloroquine significantly reduced MB49 tumor growth (Figure 8A) and tumor size (Figure 8B,C) and prolonged survival (Figure 8D) in both control and PD‐L1^KO^ tumors. However, PD‐L1^KO^ MB49 tumors were significantly more sensitive to combination therapy compared to control MB49 cells in each respect (Figure 8C,D).

## 4 DISCUSSION

Prior work from our group and others has shown that tumor cell‐intrinsic PD‐L1 mediates important pathologic signals in melanoma, ovarian, breast, lung, and colon cancer cells, independent of its well‐appreciated tumor‐extrinsic T‐cell interactions. Tumor cell‐intrinsic PD‐L1 signals modulate mTOR, autophagy, growth, metastasis, treatment resistance (e.g., small molecules, chemotherapy, immunotherapy), epithelial‐to‐mesenchymal transition, and DNA damage repair in these various tumor cells.^12^, ^13^, ^14^, ^30^, ^31^ Nonetheless, there are no published reports of tumor cell‐intrinsic PD‐L1 signals or their effects in BC.

To study BC cell‐intrinsic PD‐L1 signals, we used CRISPR/Cas9 to knock PD‐L1 out in the well‐established mouse and human BC cell lines, MB49 and RT4, respectively. PD‐L1 controlled RT4 cell proliferation in vitro consistent with our prior report in melanoma and ovarian cancer,^13^ whereas PD‐L1 had negligible effect on MB49 cell proliferation. Differences in these tumor cells could represent species differences, distinct tumor mutational profiles, BC molecular subtype (MB49 is basal‐like, whereas RT4 is luminal‐like),^23^ or differential effects of mTORC1 on tumor proliferation in these different cell lines, among other possibilities. Nonetheless, in vitro proliferation of both RT4 and MB49 cells was reduced in vitro by α‐PD‐L1 antibody. We excluded complement‐dependent cytotoxicity as a mechanism. Differences in MB49 proliferation in response to genetic deletion of PD‐L1 versus α‐PD‐L1 antibody treatment could be related to the integration of specific signals affected by total PD‐L1 knockout versus the signal subset affected by α‐PD‐L1, or the induction of a negative PD‐L1 signal by α‐PD‐L1 that would be absent in PD‐L1^KO^ cells. These data raise the intriguing possibility that α‐PD‐L1 antibody‐induced signals could contribute to α‐PD‐L1 treatment efficacy in unexpected ways, an area of current investigation.

Control MB49 cells grew well after subcutaneous challenge into wild type mice, whereas PD‐L1^KO^ MB49 cells did not and exhibited spontaneous regressions not seen after control MB49 challenge. Nonetheless, PD‐L1^KO^ MB49 cells grew well after subcutaneous challenge into immune‐deficient NSG mice, although still significantly more slowly versus control MB49 challenged subcutaneously and producing fewer lung metastases. These data are consistent with MB49 PD‐L1 signals playing a major role in immune defenses as expected, but also consistent with PD‐L1 mediating important tumor‐intrinsic growth and metastatic signals, which could be through mTORC1, seen as almost total inhibition of the phosphorylation (activation) of the mTORC1 target molecules rpS6 and 4EBP‐1 in PD‐L1^KO^ versus control MB49 and RT4, among other possibilities.

Control human RT4 cells easily established subcutaneous tumors in NSG mice, whereas PD‐L1^KO^ RT4 cells did not grow at all in NSG mice. RT4 cell proliferation was slowed to a greater extent by genetic PD‐L1 knockout versus MB49 in vitro, suggesting that PD‐L1 growth signals have greater influence on RT4 versus MB49 cell proliferation. Finally, to distinguish between cell‐intrinsic growth effects and tumor microenvironment effects, we challenged wild‐type mice with control and PD‐L1^KO^ MB49 cells orthotopically in bladder and found that MB49 PD‐L1^KO^ tumors grew in bladder, but significantly more slowly versus control cells. Thus, PD‐L1‐dependent BC cell growth effects could have anatomic compartment‐specific effects.

Tumor cell‐intrinsic PD‐L1 exerts diverse effects on important cellular survival and signaling pathways including mTORC1 and autophagy, among others.^13^ We found that MB49 PD‐L1 increased mTORC1 signals as evidenced by phosphorylation of the downstream mTORC1 targets 4E‐BP1 and rpS6. It also increased the phosphorylation of AKT^S473^ consistent with increased mTORC2 signals, similar to our prior studies of PD‐L1 intracellular signals in the human ES2 ovarian cancer cell line.^13^ Pharmacologic mTORC1 inhibition using rapamycin was less effective in PD‐L1^KO^ versus control MB49 cells suggesting that this generally poorly effective treatment strategy might be more effective in BC tumors with low PD‐L1 expression, or paradoxically, low mTORC1 signaling.

Previous studies in osteosarcoma, head and neck, and breast cancer have shown that tumor cell‐intrinsic PD‐L1 signals mediate chemotherapy resistance.^27^, ^28^, ^29^ Control and PD‐L1^KO^ MB49 cells both experienced cytotoxicity from gemcitabine or cis‐platinum. In RT4 cells, BC cell‐intrinsic PD‐L1 conferred cis‐platinum, but not gemcitabine resistance, consistent with the concept that tumor factors in addition to PD‐L1 can dictate chemosensitivity. BC cell‐intrinsic PD‐L1 promoted both steady‐state autophagy and autophagic flux, in striking contrast with our studies in melanoma and ovarian cancer, where tumor cell‐intrinsic PD‐L1 inhibited autophagy.^13^ Together, these data provide evidence that BC cell‐intrinsic PD‐L1 modulates tumor virulence and signaling in distinct ways in distinct cancers that could influence tumor biology and treatment responses.

We considered that PD‐L1‐mediated autophagic flux could generate a therapeutic vulnerability. Accordingly, we tested the combination of cis‐platinum plus chloroquine, an autophagy inhibitor, in vivo and found that PD‐L1^KO^ MB49 tumors were significantly more sensitive versus control MB49 tumors to combination therapy. While combination therapy with gemcitabine plus cis‐platinum is standard‐of‐care in the clinical treatment of muscle‐invasive BC,^32^ and chloroquine has been tested as an anticancer agent in human trials,^33^, ^34^ BC treatment with chloroquine has only been evaluated in preclinical studies, where it enhances BC radiosensitivity^35^ and induces BC cell apoptosis in vitro.^36^ Though many mechanisms of cis‐platinum resistance in BC have been proposed,^37^ understanding remains incomplete and PD‐L1 effects deserve additional study in this regard.

In summary, we identified previously unrecognized BC cell‐intrinsic PD‐L1 signals mediating important pathologic signals including mTORC1 activation, growth promotion, and chemotherapy resistance. These studies provide insights into potential immune‐independent effects of existing BC immunotherapies targeting PD‐L1, or that pharmacologic or genetic PD‐L1 depletion could improve treatment responses to specific agents and suggest treatment response biomarkers. We also provide evidence for how tumor PD‐L1 can affect the efficacy of commonly used BC chemotherapies and provide preclinical evidence for the use of novel treatment combinations to improve BC treatments.

## Conflicts of interests

The authors have no conflicting financial interests to declare.

## Supporting information

Fig S1

Fig S2

## Data Availability

The datasets used and/or analyzed during the current study are available from the corresponding author upon reasonable request.
