# Supplementary material for: Bladder cancer cell‐intrinsic PD‐L1 signals promote mTOR and autophagy activation that can be inhibited to improve cytotoxic chemotherapy
Source: Cancer Med. 2021 Feb 24;10(6):2137–52. doi: 10.1002/cam4.3739 (PMC7957205; doi:10.1002/cam4.3739)
Supplement: Supplementary file 1 — Fig S1 [file CAM4-10-2137-s001.pdf]

Supplementary Fig 1

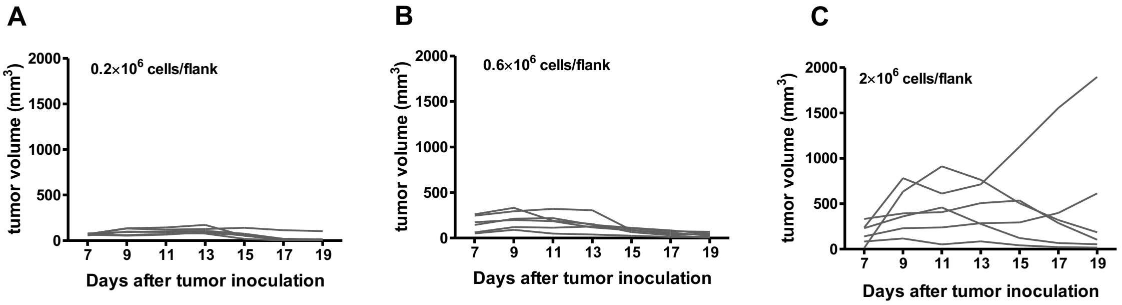

Supplementary Figure 1. In vivo growth failure of PD-L1^{KO} MB49 bladder cancer cells. *In vivo* challenge of 0.2 x 10^6 (A), 0.6 x 10^6 (B), and 2 x 10^6 (C) cells PD-L1^{KO} MB49 cells (clone 18) all failed to reliably produce SQ tumors in WT C57Bl6 mice.
