# Supplementary material for: Bladder cancer cell‐intrinsic PD‐L1 signals promote mTOR and autophagy activation that can be inhibited to improve cytotoxic chemotherapy
Source: Cancer Med. 2021 Feb 24;10(6):2137–52. doi: 10.1002/cam4.3739 (PMC7957205; doi:10.1002/cam4.3739)
Supplement: Supplementary file 2 — Fig S2 [file CAM4-10-2137-s002.pdf]

Supplementary Figure 2

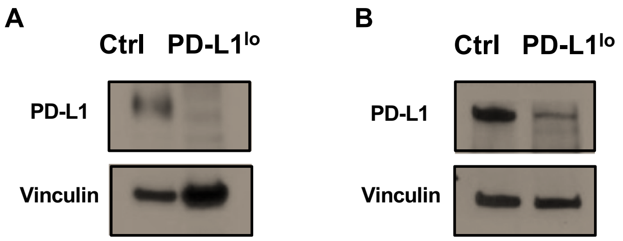

Supplementary Figure 2. Generation of PD-L1^{lo} UM-UC-3 and UM-UC-14 BC cells. (A) PD-L1 expression is reduced by 96.22% in PD-L1^{lo} UM-UM-3 cells. (B) PD-L1 expression is reduced by ~75% in UM-UC-14 BC cells.
